# Presentation and Real-World Management of Giant Cell Arteritis (Artemis Study)

**DOI:** 10.3389/fmed.2021.732934

**Published:** 2021-11-11

**Authors:** Alfred Mahr, Eric Hachulla, Hubert de Boysson, Nassim Guerroui, Emmanuel Héron, Stéphane Vinzio, Jonathan Broner, François-Xavier Lapébie, Martin Michaud, Laurent Sailler, Thierry Zenone, Mohamed Djerad, Mathieu Jouvray, Emilie Shipley, Nathalie Tieulie, Guillaume Armengol, Bastien Bouldoires, Jean-Francois Viallard, Isabelle Idier, Marc Paccalin, Valérie Devauchelle-Pensec

**Affiliations:** ^1^Department of Internal Medicine, University Hospital Paris (AP-HP, Saint Louis), Paris, France; ^2^Department of Internal Medicine, Lille University Hospital, Lille, France; ^3^Department of Internal Medicine, Caen University Hospital, Caen, France; ^4^Department of Rheumatology, European Hospital of Marseille, Marseille, France; ^5^Department of Internal Medicine, Hospital Quinze-Vingts, Internal Medicine, Paris, France; ^6^Department of Internal Medicine, Groupe Hospitalier Mutualiste de Grenoble, Grenoble, France; ^7^Department of Internal Medicine, University Hospital Centre Nimes, Nimes, France; ^8^Department of Internal Medicine, University Hospital Centre Toulouse, Toulouse, France; ^9^Department of Internal Medicine, Hopital Joseph Ducuing Toulouse, Toulouse, France; ^10^Department of Internal Medicine, University Hospital of Toulouse, Toulouse, France; ^11^Department of Internal Medicine, General Hospital of Valence, Valence, France; ^12^Department of Internal Medicine, General Hospital of Nevers, Nevers, France; ^13^Department of Internal Medicine, General Hospital of Arras, Arras, France; ^14^Department of Rheumatology, General Hospital of Dax, Dax, France; ^15^Department of Rheumatology, University Hospital of Nice, Nice, France; ^16^Department of Internal Medicine, Rouen University Hospital, Rouen, France; ^17^Department of Internal Medicine, Civil Hospital of Colmar, Colmar, France; ^18^Department of Internal Medicine, University Hospital of Bordeaux, Bordeaux, France; ^19^Medical Affairs, Chugai Pharma France, Paris La Défense, Paris, France; ^20^Department of Internal Medicine, University Hospital of Poitiers, Poitiers, France; ^21^Rheumatology Department, University Hospital La Cavale Blanche, Brest, France

**Keywords:** giant cell arteritis, phenotype [mesh], management - healthcare, observational, glucocorticoids (GCs), methotrexate, tocilizumab

## Abstract

**Background:** Few studies of daily practice for patients with giant cell arteritis (GCA) are available. This French study aimed to describe the characteristics and management of GCA in a real-life setting.

**Methods:** Cross-sectional, non-interventional, multicenter study of patients ≥50 years old who consulted hospital-based specialists for GCA and were under treatment. Patient characteristics and journey, diagnostic methods and treatments were collected. Descriptive analyses were performed.

**Results:** In total, 306 patients (67% females, mean age 74 ± 8 years old) were recruited by 69 physicians (internists: 85%, rheumatologists: 15%); 13% of patients had newly diagnosed GCA (diagnosis-to-visit interval <6 weeks). Overall median disease duration was 13 months (interquartile range 5–26). Most patients were referred by general practitioners (56%), then ophthalmologists (10%) and neurologists (7%). Most common comorbidities were hypertension (46%), psychiatric disorders (10%), dyslipidemia (12%), diabetes (9%), and osteoporosis (6%). Initial GCA presentations included cranial symptoms (89%), constitutional symptoms (74%), polymyalgia rheumatica (48%), and/or other extra-cranial manifestations (35%). Overall, 85, 31, 26, and 30% of patients underwent temporal artery biopsy, high-resolution temporal artery Doppler ultrasonography, ^18^FDG-PET, and aortic angio-CT, respectively. All patients received glucocorticoids, which were ongoing for 89%; 29% also received adjunct medication(s) (methotrexate: 19%, tocilizumab: 15%). A total of 40% had relapse(s); the median time to the first relapse was 10 months. Also, 37% had comorbidity(ies) related to or aggravated by glucocorticoids therapy.

**Conclusion:** This large observational study provides insight into current medical practices for GCA. More than one third of patients had comorbidities related to glucocorticoid therapy for a median disease duration of 13 months. Methotrexate and tocilizumab were the most common adjunct medications.

## Key Messages

- Large-vessel giant cell arteritis (i.e., large-vessel involvement only) is rare (5%).
- 37% of patients experienced at least one comorbidity related to or aggravated by glucocorticoids treatment.
- One third of patients received adjunctive medication(s) (methotrexate, tocilizumab).

## Introduction

Giant cell arteritis (GCA) is an inflammatory vasculopathy and the most frequent systemic vasculitis in Western countries. GCA involves large- and medium-sized arteries, predominantly the extracranial branches of the carotid arteries and the subclavian and axillary branches of the aorta (1). GCA affects older people and women more than men, with an incidence of 10 to 20/100,000 people ≥50 years old in Europe (2).

In addition to the classic cranial arteritis features, GCA includes polymyalgia rheumatica (PMR), other extra-cranial manifestations, and/or constitutional symptoms (3, 4). Apart from upper- or lower-limb claudication, large-vessel GCA (LV-GCA) might be asymptomatic. All these presentations may coincide together, occur as independent clinical subsets, or overlap. Visual ischemic complications, stroke, and aortic aneurysm or dissection are the most feared complications.

The diagnostic methods and recommended management of GCA have recently evolved. For a few decades, temporal artery biopsy (TAB) has been considered the gold standard for GCA diagnosis, and it often remains the first-intention test to propose, notably in France (5, 6). However, less invasive vascular imaging modalities are increasingly being used to study the cranial or extracranial arteries, including aorta inflammation. In this context, recommendations of the European League against Rheumatism (EULAR) on imaging in LV-GCA (7) were updated in 2018. In particular, EULAR recommendations now promote ultrasonography as the first choice for diagnosis in predominantly cranial GCA, with an additional investigation, including TAB, when the diagnosis is still in question.

Glucocorticoids (GCs) are the treatment of choice for GCA and should be initiated immediately with suspected GCA to induce remission and prevent complications (8); however, relapses are common, up to 40% (6), when the GC dose is tapered, which leads to prolonged or repeated oral treatment with risk of adverse effects (9). According to recent European or French recommendations (6, 8), tocilizumab (TCZ) (10, 11) or alternatively methotrexate (MTX) can be combined with GCs to reduce the GC toxicity. Other potential adjunct therapies (immunosuppressants and biologics) lack convincing results (6).

We do not know to what extent recent recommendations on the diagnosis and treatment of GCA are implemented in clinical practice. In France, first responses have been provided in a recent study based on national administrative health insurance claims data (12): TAB was used in 51% of the patients, and MTX was the most prescribed GC-sparing agent (12%). However, we have limited data on patients' comorbidities, clinical presentation and forms, cumulative doses for GCs as well as the use of imaging techniques for GCA diagnosis.

The aim of the study was to provide an overview of GCA, specifically the characteristics and management, in a real-life setting.

## Materials and Methods

### Study Design

ARTEMIS is a cross-sectional, non-interventional, multicenter French study conducted among hospital-based internists and rheumatologists. All 2,676 eligible specialists, hospital-based internists and rheumatologists in a national independent French database were invited to participate in the study. Each specialist who agreed to participate was requested to include, consecutively during the inclusion period, a maximum of 10 patients to limit a potential center effect. According to French legislation regarding non-interventional studies, the ARTEMIS protocol (ClinicalTrials.gov NCT03658889) was approved by the ethics committee (authorization no: 2018-A00841-54. 2-18-37), which guarantees confidentiality to the participants. All patients were informed with an information document completed by investigators about the study before enrolment and had no objection to sharing their data (written consent is not mandatory for non-interventional studies according to French legislation: CNIL1818705X, No 2018-15). This study was performed in accordance with the Helsinki Declaration of 1964 and its later amendments.

### Patients and Data Collection

Eligible patients were adults ≥50 years old who were seen as in- or outpatients for a new or previously diagnosed GCA. The diagnosis of GCA was according to the investigator's judgment regardless of the specific criteria used. In addition, patients had to be under GCA treatment at the inclusion visit and have no objection to participate in the study. Patients participating in an interventional study were excluded.

At the study visit, specialists collected the following information by using an electronic Case Report Form: patient journey, GCA characteristics, diagnostic methods, GCA treatments and comorbidities related to or aggravated by GCs. GCA activity was assessed by using a 100-mm visual analog scale (VAS) completed by patients and physicians.

### Statistics

No formal sample size was calculated for this non-interventional study. However, from a previous study of GCA patients (database analysis of the *Echantillon Généraliste des Bénéficiaires*), there are 2,300 incident patients/year in France (12). Therefore, the inclusion of 300 incident and prevalent patients during the planned 5-month recruitment period seemed realistic.

Descriptive analyses were performed. Study variables were assessed with mean ± standard deviation (SD) or median [interquartile range (IQR)] for continuous variables and number (%) for categorical variables. Missing values were not replaced. Statistical analysis involved using SAS 9.3 (SAS Institute, Cary, NC, USA).

The GCA diagnosis was considered early, standard, or late when the time between first GCA symptoms and diagnosis was <1 month, 1–3 months, or >3 months, respectively. Analysis of GCA data was according to the diagnosis-to-visit interval (incident disease: <6 weeks, prevalent disease: ≥6 weeks). Cranial involvement included headaches, temporal artery abnormalities, jaw claudication, scalp tenderness, visual symptoms, and stroke and transient ischemic attack(s); LV involvement included aortic aneurysm or dilatation, aortitis and/or involvement of aortic branch(s) on imaging, claudication of a limb, sign(s) of subclavian stenosis.

## Results

### Participant Physicians and Patient Disposition

Of the 2,676 French eligible specialists invited to participate in the study, 69 from 53 centers accepted and included at least one eligible patient. Participating specialists were mainly working in a university hospital (39/69, 56.5%) or a general hospital (30/69, 43.5%).

Of the 308 patients included from August to November 2018 by 69 hospital-based specialists (internists: 84%, rheumatologists: 15%, geriatricians: 1%), 306 fulfilled all the selection criteria. Two patients without GCA treatment at the study visit were excluded.

### Characteristics of Patients

The characteristics of the patients are detailed in Table 1. The mean age of patients (67% female) was 74.0 ± 7.9 years at the study visit. Most (83%) patients had comorbidity(ies) before GCA was diagnosed, mainly hypertension, dyslipidemia, diabetes, and/or osteoporosis. Eye disorders (cataract and glaucoma) were reported in 10% of patients.

**Table 1:** Characteristics of patients.

**Parameter**	**Number of analyzed patients**	**Total *(N = 306)***
**Demographics at inclusion**		
Age (years), mean ± SD	306	74.0 ± 7.9
Age ≥70 years, *n* (%)	306	222 (72.5)
Female sex, *n* (%)	306	206 (65.3)
**Body mass index at diagnosis** (kg/m^2^)	292	
Mean ± SD		24.6 ± 4.0
>25 kg/m^2^		125 (42.8%)
**Smoking status at inclusion**, *n* (%)	279	
Non-smoker ever		197 (70.6)
Former smoker		57 (20.4)
Smoker		25 (9.0)
**At least one comorbidity prior to GCA diagnosis**, *n* (%)	306	253 (82.7)
**Comorbidities (≥2% of patients)**, *n* (%)	306	
Hypertension		140 (45.8)
Dyslipidemia		36 (11.8)
Diabetes mellitus		29 (9.5)
Cataract		19 (6.2)
Osteoporosis		18 (5.9)
Depression		17 (5.6)
Atrial fibrillation		16 (5.2)
Hypothyroidism		15 (4.9)
Hypercholesterolemia		12 (3.9)
Glaucoma		12 (3.9)
Breast cancer		10 (3.3)
Myocardial ischemia		9 (2.9)
Asthma		9 (2.9)
Sleep apnea syndrome		8 (2.6)
Osteoarthritis		8 (2.6)
Polymyalgia rheumatica		8 (2.6)
Cerebrovascular accident		7 (2.3)
**Time between GCA diagnosis and inclusion (months), mean ± SD**	306	21.0 ± 26.4
**Concomitant treatments at GCA diagnosis**, *n* (%)		
Antihypertensive agent	305	141 (46.2)
Antiplatelet agent	304	68 (22.4)
Proton pump inhibitor	305	67 (22.0)
Statin	305	40 (13.1)

*GCA, giant cell arteritis; IQR, interquartile range; SD, standard deviation*.

At the study visit, most patients (79%) consulted the participant hospital-based specialists as outpatients, and most (87%) had prevalent disease (diagnosis-to-visit interval >6 months). Various physicians referred the patients to participant specialists, mainly general practitioners (56%), followed by ophthalmologists (10%), neurologists (7%), emergency physicians (6%), rheumatologists (5%), or internists (4%). Since the first GCA symptom, patients consulted a mean of 2.1 ± 1.2 specialists (general practitioners: 85%, ophthalmologists: 29%, neurologists: 12%, emergency doctors: 18%, internists: 19%, rheumatologists: 14%).

### Initial Patient Presentation

At the study visit, the median time since GCA diagnosis was 13.0 months (IQR 5.0–26.0): 15.0 months (7.0–30.0) for patients with prevalent disease and 0.6 months (0.2–1.0) for those with a new diagnosis. Overall, 21% of patients had an early diagnosis, 57% a diagnosis within the standard timeframe, and 22% a late diagnosis.

**Figure 1:**
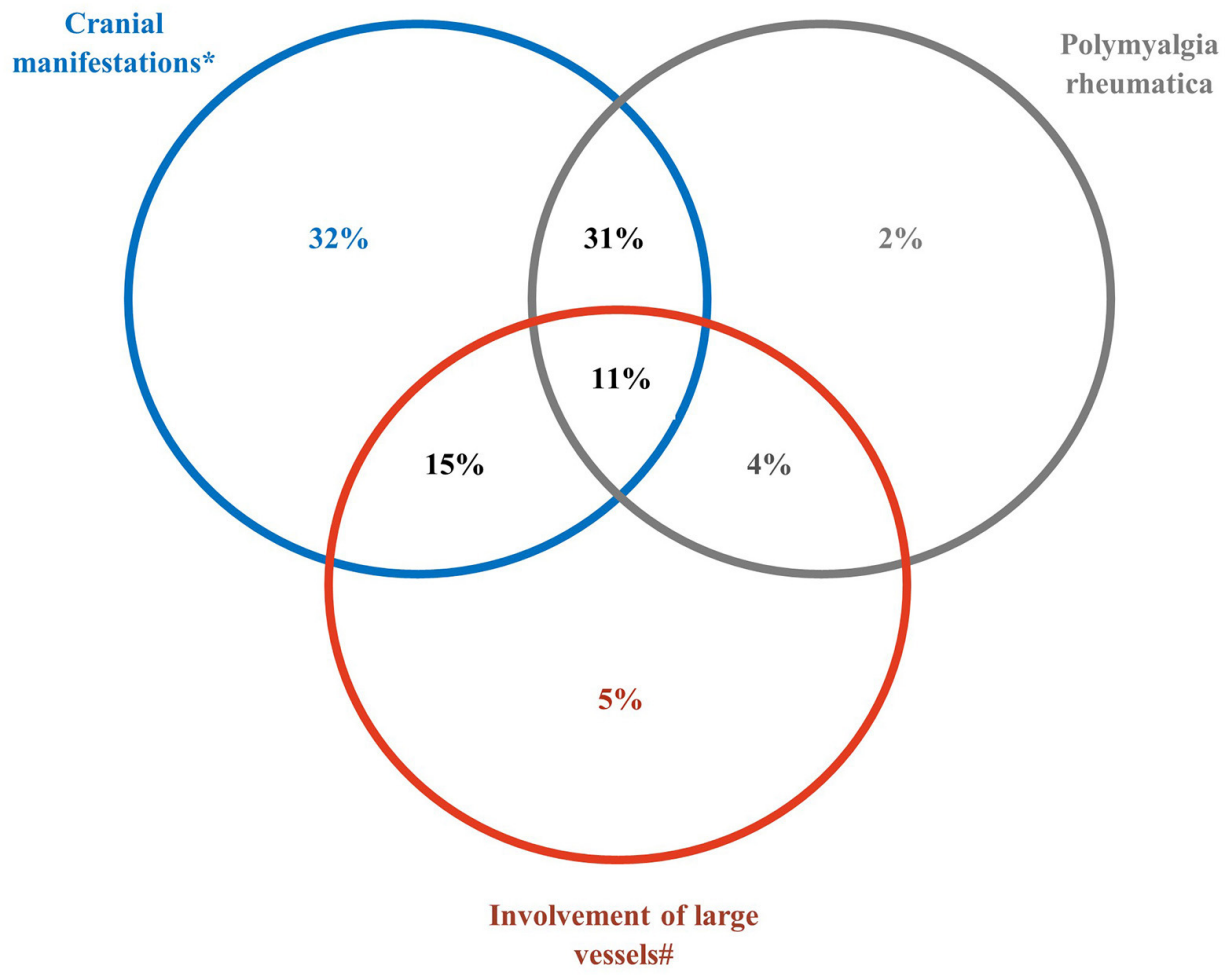
Clinical phenotypes of giant cell arteritis at diagnosis. *The subset of cranial manifestations includes patients with strokes and transient ischemic attack(s). ^#^The subset of large vessel involvement includes aortic aneurysm or dilatation, aortitis and/or involvement of aortic branch(s) in imaging, claudication of a limb, sign(s) of subclavian stenosis.

In total, 271 (89%) patients had cranial involvement; 29 (9.5%) had an anterior ischemic optic neuropathy, 29 (9.5%) diplopia, and 5 (1.6%) blindness. Cranial involvement was associated with PMR in 42% of patients and with LV involvement in 26%. Isolated PMR and PMR associated with LV-GCA were diagnosed in 6% of patients. In all, 5% of patients had LV-GCA (i.e., LV involvement only) (Figure 1). The GCA characteristics at diagnosis are detailed in Table 2.

**Table 2:** Initial presentation of giant cell arteritis.

**Variable at diagnosis**	Incident disease^*^N = 39 (%)	Prevalent disease^*^			**All patients *N = 306 (%)***
		***No relapse N = 145 (%)***	***≥1 relapse N = 122 (%)***	***Total N = 267 (%)***	
**Cranial manifestations**, *n* (%)	**33/39 (84.6)**	**132/145 (91.0)**	**106/122 (86.9)**	**238/267 (89.1)**	**271/306 (88.6)**
Headaches	30 (90.9)	110 (83.3)	84 (88.7)	204 (85.7)	234 (86.3)
Scalp sensitivity	18 (54.5)	55 (41.7)	62 (58.5)	117 (49.2)	135 (49.8)
Anomalies of the temporal arteries	13 (39.4)	68 (51.5)	50 (47.2)	118 (49.6)	131 (48.3)
Anterior ischemic optic neuropathy	2 (5.1)	21 (14.5)	6 (4.9)	27 (10.1)	*29 (9.5)*
Diplopia	5 (12.8)	12 (8.3)	12 (9.8)	24 (9.0)	*29 (9.5)*
Mouth pain or jaw claudication during mastication	18 (54.5)	65 (49.2)	49 (46.2)	114 (47.9)	132 (48.7)
Stroke or transient ischemic attack	1 (3.0)	12 (9.1)	3 (2.8)	15 (6.3)	16 (5.9)
Neck pain	1 (3.0)	3 (2.3)	2 (1.9)	5 (2.1)	6 (2.2)
Other	3 (9.1)	4 (3.0)	3 (2.8)	7 (2.9)	10 (3.7)
**PMR symptoms**, *n* (%)	**24/39 (61.5)**	**66/145 (45.5)**	**58/122 (47.5)**	**124/267 (46.4)**	**148/306 (48.4)**
Morning stiffness and/or pain in the shoulder girdle	21 (87.5)	53 (80.3)	52 (89.7)	105 (84.7)	126 (85.1)
Morning stiffness and/or pains in the pelvic girdle	11 (45.8)	34 (51.5)	37 (63.8)	71 (57.3)	82 (55.4)
Inflammatory arthromyalgia	12 (50.0)	38 (57.6)	42 (72.4)	80 (64.5)	92 (62.2)
Peripheral arthritis	4 (16.7)	9 (13.6)	7 (12.1)	16 (12.9)	20 (13.5)
Arthralgia	0 (0.0)	4 (6.1)	0	4 (3.2)	4 (2.7)
Other	2 (8.3)	5 (7.6)	2 (3.4)	7 (5.6)	9 (6.1)
**Extracranial events (excluding PMR)**, *n* (%)	**8/39 (20.5)**	**44/145 (30.3)**	**56/122 (45.9)**	**100/267 (37.5)**	**108/306 (35.3)**
Thoracic or abdominal aortic aneurysm and/or dilatation	1 (12.5)	8 (18.2)	5 (8.9)	13 (13.0)	14 (13.0
Aortitis and-or involvement of aortic branch(s) in imaging	6 (75.0)	36 (81.8)	30 (53.6)	66 (66.0)	72 (66.7)
Angina and-or myocardial infraction	0 (0.0)	0	3 (5.4)	3 (3.0)	3 (2.8)
Claudication of an upper and/or lower limb	2 (25.0)	8 (18.2)	9 (16.1)	17 (17.0)	19 (17.6)
Sign(s) of subclavian stenosis	1 (12.5)	6 (13.6)	6 (10.7)	12 (12.0)	13 (12.0)
Other	0 (0.0)	5 (11.4)	11 (19.6)	16 (16.0)	16 (14.8)
**ESR (mm/1**^**st**^ **h) >50 mm/h**, *n* (%)	**19/26 (73.1)**	**58/80 (72.5)**	**52/62 (83.9)**	**110/142 (77.5)**	**129/168 (76.8)**
**CRP (mg/L) >25 mg/L**, *n* (%)	**27/36 (75.0)**	**118/139 (84.9)**	**98/113 (86.7)**	**216/252 (85.7)**	**243/288 (84.4)**
**General signs**, *n* (%)	**25/39 (64.1)**	**111/145 (76.6)**	**91/122 (74.6)**	**202/267 (75.7)**	**227/306 (74.2)**
Fever >38°C	11 (44.0)	44 (39.6)	45 (50.0)	89 (44.3)	100 (44.2)
Weight loss	14 (56.0)	64 (57.7)	51 (46.0)	115 (56.9)	129 (56.8)
Alteration of the general condition	24 (96.0)	94 (84.7)	79 (86.8)	173 (85.6)	197 (86.8)

*CRP, C-reactive protein; ESR, erythrocyte sedimentation rate; PMR, polymyalgia rheumatica*.

* *Incident patients: diagnosis-to-visit interval <6 weeks; prevalent patients: diagnosis-to-visit interval ≥6 weeks. Bold values indicate proportion of patients in each group with available data*.

Cranial manifestations at diagnosis were more frequent in patients with early and standard-timeframe diagnoses (92% for both timeframes *vs*. 82% with late diagnosis). By contrast, patients with a late diagnosis more frequently had PMR symptoms (65 *vs*. 43–47%) and extracranial manifestations (49 *vs*. 27–31%).

Among patients with prevalent disease, those with at least one relapse initially experienced extracranial event(s) (excluding PMR) more often than those without any relapse since diagnosis (46 *vs*. 30%, *p* = 0.009). At the study visit, GCA activity assessed on a 100-mm VAS was higher for patients with incident than prevalent disease [median 27 (IQR 6–63) vs. 18 (5–47)] but the difference did not reach statistical significance (*p* = 0.125). GCA activity rated by physicians was significantly lower for patients with prevalent disease than patient with incident disease [median 3 (IQR 0–12) and 10 (2–53), *p* = 0.0015], and also showed smaller numbers compared with patient ratings.

### Diagnostic Methods

**Figure 2:**
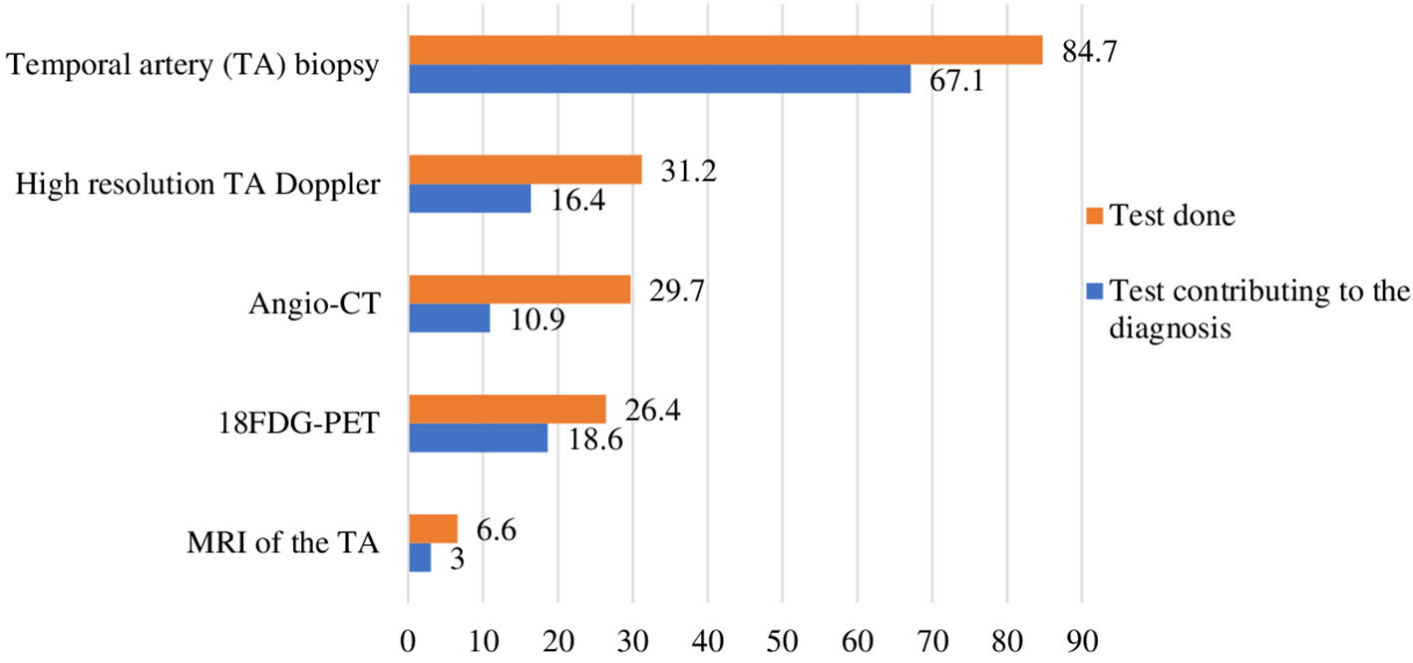
Diagnostic tests for giant cell arteritis. Proportion of patients (%). CT, computed tomography; MRI, magnetic resonance imaging; ^18^FDG-PET, ^18^F-fluorodesoxyglucose positron emission tomography.

GCA diagnosis can be based on clinical symptoms and physical examination, acute phase reactants, TAB, and/or imaging (high-resolution color Doppler ultrasonography, MRI of the temporal arteries, angio-CT or ^18^FDG-PET). The mean number of methods used for GCA diagnosis was 1.9 ± 1.1. The methods used for GCA diagnosis and their contribution to the diagnosis are presented in Figure 2. Overall, TAB was the most frequently used technique (85%). High-resolution temporal artery Doppler ultrasonography, ^18^FDG-PET and aortic angio-CT were also frequently performed, in 31, 26 and 30% of patients, respectively, whereas MRI of the temporal arteries was used in 7% of patients. Overall, TAB confirmed the diagnosis for 54.5% of patients, high-resolution temporal artery Doppler for 15.6%, ^18^FDG-PET for 17%, aortic angio-CT for 8.8% and MRI for 3%. When performed, ^18^FDG-PET most often established the diagnosis of GCA, in 70% of patients vs. 67% for TAB. In addition, ^18^FDG-PET was more often used than TAB for patients with LV involvement (73 *vs*. 67%) (Supplementary Table 1). The use of vascular imaging was more frequent in patients with a late than early diagnosis (>3 *vs*. <1 month): high-resolution temporal artery Doppler ultrasonography: 42 *vs*. 26% of patients; ^18^FDG-PET: 46 *vs*. 17%; angio-CT: 35 *vs*. 19%.

### Relapses and Complications

Patients with prevalent disease had experienced at least one relapse after diagnosis (46%), and most had one or two relapses (57 and 26%, respectively). The median time to first relapse was 10 months (IQR 5–19). Relapses were mainly evaluated according to clinical criteria (81%) and/or laboratory criteria of elevated levels of acute phase reactants (81%); they were rarely evaluated according to only laboratory criteria (14%). Cranial and rheumatic symptoms were the most frequent clinical criteria reported (in 52 and 44% of patients, respectively).

According to investigators' judgement, 16% of patients had at least one GCA complication since diagnosis, which was mainly ophthalmic (5%), psychiatric (3%) and or vascular (3%).

### Treatment of GCA

All patients received GCs at least once after diagnosis, and GC therapy was ongoing in 89% at the study visit (Table 3). At diagnosis, intravenous pulse GCs were given to 54 (16%) patients for a median of 3 days (IQR 3–3). The median dose of GCs was higher for patients with incident than prevalent disease [40 (IQR 30–50) *vs*. 8 (5–15) mg/day]. Overall, the median cumulative oral GC dose, assessed in 87 patients, was 4,305 mg (IQR 1,920–7,000); the median cumulative oral GC dose, assessed in the 74 patients with prevalent disease, was 4,985 mg (IQR 2,838–7,170). For the 21 patients with relapse and with available data, the median cumulative dose was 7,400 mg (IQR 4,867–9,435). Most relapses (80%) were diagnosed in patients with ongoing GC therapy [median dose 10 mg/day (IQR 5–17)]; 11 and 3% of relapses occurred under ongoing immunosuppressive therapy or therapy with targeted biologics, respectively. Relapses were treated with GCs at a median prednisone equivalent dose of 20 mg/day (IQR 10–30) in 95% of cases, immunosuppressants in 21% and/or targeted biologics in 19%.

**Table 3:** Glucocorticoids for giant cell arteritis since diagnosis.

**Glucocorticoids (GCs)**	**Number of analyzed patients**	Incident disease^*^(N = 39)	Prevalent disease^*^			**All patients *(N = 306)***
			***No relapse (N = 145)***	***≥1 relapse (N = 122)***	***Total (N = 267)***	
Number of GC courses, *n* (%)	306					
1		39 (100.0)	142 (97.9)	94 (77.0)	236 (88.4)	275 (89.9)
2		0 (0.0)	3 (2.1)	26 (21.3)	29 (10.9)	29 (9.5)
3		0 (0.0)	0 (0.0)	2 (1.6)	2 (0.7)	2 (0.7)
GC course duration (months), median (IQR)	306	1.00 (0.00–1.00)	10.00 (4.00–16.00)	20.00 (11.50–32.50)	14.00 (7.00–25.00)	12.50 (5.00–23.00)
Ongoing treatment with GCs at study visit (mg), *n* (%)	306	39 (100.0)	131 (90.3)	103 (84.4)	234 (87.6)	273 (89.2)
Current dose of GCs at study visit (mg), median (IQR)	273	40.00 (30.00–50.00)	9.00 (5.00–15.00)	8.00 (5.00–12.00)	9.00 (5.00–15.00)	9.00 (5.00–20.00)
Total cumulative oral dose of GCs (mg), median (IQR)	87	1080.00 (660.00–1800.00)	4350.00 (2580.00–5670.00)	7400.00 (4867.00–9435.00)	4985.00 (2838.00–7170.00)	4305 (1920–7000)

*IQR, interquartile range*.

* *Incident disease: diagnosis-to-visit interval <6 weeks; prevalent disease: diagnosis-to-visit interval ≥6 weeks*.

In addition, 29% of patients were receiving or received at least one adjunct treatment for GCA [immunosuppressants (19%) and/or targeted biologic agents (16%)], and 6% two different adjunct medications (Table 4). MTX and TCZ were the most frequently prescribed adjunct medications (19 and 15% of patients, respectively). For the 25 (8%) patients who stopped MTX before the study visit, the mean treatment duration was 16.8 ± 15.7 months. The current mean dose of MTX was 14.4 ± 4.8 mg/week for the 35 (11%) patients who were receiving the drug at inclusion. For the 9 (3%) patients who stopped TCZ before the study visit, the mean treatment duration was 21.9 ± 16.6 months. Other adjunct medications, namely azathioprine, cyclophosphamide, leflunomide, infliximab or adalimumab, were rarely prescribed (Table 4).

**Table 4:** Adjunctive treatments for giant cell arteritis since diagnosis.

**Variable**	**Number of analyzed patients**	**Total *N = 306***
**At least one adjunct treatment**, ***n*** **(%)**	**306**	**90 (29.4)**
Immunosuppressants, *n* (%)	306	59 (19.3)
Methotrexate		58 (18.9)
Azathioprine		2 (0.7)
Cyclophosphamide		1 (0.3)
Leflunomide		1 (0.3)
Targeted biologic agents, *n* (%)	306	48 (15.7)
Adalimumab		1 (0.3)
Infliximab		1 (0.3)
Tocilizumab		47 (15.4)

Overall, 37% of patients experienced at least one comorbidity related to or aggravated by the GCs use, mainly diabetes (12%), hypertension (10%), osteopenia/osteoporosis/osteoporotic fractures (7%), insomnia (3%), and infections (3%). Cataract and glaucoma were reported as GC-related events in 1% of patients for both events (Supplementary Table 2). Osteoporosis treatment and calcium-vitamin D were given to 47 and 37% patients, respectively, in the period following diagnosis of GCA and 61% received antiplatelet agents.

## Discussion

In the context of the rapidly evolving landscape of recommendations for managing GCA, this French study provides insights into current medical practices in hospital centers for GCA (GCA subtypes, patient pathway, diagnostic methods, and GCA treatments).

Overall, 306 patients under treatment for GCA were enrolled by 69 hospital-based specialists from 53 centers in 2018. Most patients were females and most were at least 70 years old at the study visit, in accordance with the well-known characteristics of GCA (12–15). General practitioners referred half of the patients to the specialists who participated in the study.

At initial presentation, cranial manifestations (isolated or not) were predominant (89% of patients), as expected and previously reported (14). Isolated LV-GCA was diagnosed in only 5% of patients. Delayed diagnosis was still common (>3 months after the first medical event in 22% of patients), in particular for patients with PMR symptoms or extracranial events.

Probably in line with the common cranial manifestations of GCA and headache, TAB remained the most commonly performed diagnostic test for GCA and was used in 85% of patients. This proportion was consistent with the proportion from a French retrospective study (91%) conducted in two hospital centers (13) but much higher than that reported in a study (51%) based on national administrative health insurance claims data between 2007 and 2015 (12). This difference may be explained by the fact that the ARTEMIS study did not enroll patients exclusively seen by community physicians (office-based rheumatologists or general practitioners who could have practices different from hospital-based specialists). Also, vascular imaging modalities were frequently used (from 26 to 31% of patients depending on the imaging performed) and contributed to the diagnosis, in particular for patients with extracranial manifestations. This observation may reflect a shift toward imaging techniques for GCA diagnosis, in accordance with recent European recommendations (7, 8). However, the use of large-vessel imaging was not systematic, which could explain the small proportion (11%) of patients with a diagnosis of non-cranial GCA.

Our study showed a high proportion of patients with GCA relapse(s) since diagnosis (46%), with a median time to first relapse of 10 months. These results are consistent with previous findings from a French monocentric study showing 52% relapse after a median of 12 months after diagnosis (15) and with the proportion of relapsing patients (42%) in a meta-analysis of non-interventional studies (16). In addition, we observed a significantly higher proportion of relapsing patients who presented extra-cranial event(s) at GCA diagnosis as compared with patients with no relapse during follow-up. In a retrospective monocentric French study, LV-GCA was found as an independent factor of relapse (hazard ratio 1.49, 95% confidence interval 1.002–2.12; *p* = 0.04) (15).

The toxicity related to GCs depends on both the daily dose and cumulative dose (14, 17). In our study, the high median GC dose of patients with incident disease (40 mg/day) was consistent with the median starting dose of GCs analyzed from a US database (50 mg/day) as well as the cumulative GC dose (4,305 and 4,800 mg, respectively) (14). This GC dose of patients with incident disease in our study is somewhat lower than the mean initial dose prescribed in a French population-based study (mean 41.7 *vs*. 54.5 mg/day) (18). As compared with non-hospital physicians, hospital specialists may prescribe lower prednisone doses in non-complicated GCA, the most frequent form of the disease.

Overall, after a median GCA duration of 13 months (15 months for prevalent patients), 37% of patients experienced at least one comorbidity related to or aggravated by the GCs taken (mainly diabetes, hypertension, osteopenia/osteoporosis/osteoporotic fractures, insomnia, and infections). The lower occurrence of side effects linked to the use of GCs in our study compared to the much higher previously reported figures (9), may be related to a lower cumulative dose and a better management of corticosteroids tolerance during the last decades but is still an issue in these older patients.

Overall, 29% of the studied patients received at least one adjunct agent since GCA diagnosis. MTX and TCZ were the most-prescribed GC-sparing agents (19 and 15% of patients, respectively). The doses of MTX used (mean 14.4 ± 4.8 mg/week) were in agreement with or close to recommendations, the minimum recommended dose being 15 mg/week for EULAR and from 7.5 to 15 mg/week for French recommendations (6, 7). By comparison, regarding the proportions of patients receiving an adjunct treatment, the French study based on national administrative health insurance claims data showed a slightly lower proportion of patients receiving MTX between 2007 and 2015 (12%) and no patients receiving TCZ during this period (12). The new prescriptions of TCZ observed in 2018 should be seen in relation to the recent approval of TCZ in this indication (in 2017).

Our real-world data are based on a large sample of patients with GCA defined as per physician judgement and without imposed classification criteria. Thus, the study provides findings for GCA management based on usual medical practices. The limitations of our study are inherent to its non-interventional design and that studied variables were analyzed only when available in patients' medical files. In addition, only GCA patients under treatment at the study visit had to be included, which may have led to an increased proportion of patients with long-standing therapies. Finally, because the study did not enroll GCA patients exclusively seen by community physicians, the extrapolation of our findings to other populations is cautioned.

In conclusion, this large observational study conducted in patients with recently diagnosed GCA provides insight into current medical practices for GCA in France. Our data show that non-cranial GCA remains a rare clinical phenotype of the disease despite the increasing use of LV imaging. In addition, the substantial proportion of patients with relapsing disease was confirmed, with high cumulative GC doses and adjunct medications (mainly MTX and TCZ) in one third of patients.

## Data Availability Statement

The original contributions presented in the study are included in the article/Supplementary Material, further inquiries can be directed to the corresponding author.

## Ethics Statement

The studies involving human participants were reviewed and approved by Ethics Committee (authorization No: 2018-A00841-54. 2-18-37)_Agence régionale de Santé Toulouse/France. Written informed consent for participation was not required for this study in accordance with the national legislation and the institutional requirements.

## Author Contributions

AM, EH, II, MP, and VD-P contributed to conception, design, and analyses interpretation of the study. AM organized the database. AM and II wrote the first draft of the manuscript. EH, HB, LS, and VD-P wrote sections of the manuscript. All authors contributed to manuscript revision, read, and approved the submitted version.

## Funding

This study was funded and sponsored by CHUGAI PHARMA FRANCE.

## Conflict of Interest

AM, VD-P, EH, and MP received honoraria as members of the Scientific Committee of the study. Participant physicians received fees for this study. II is a Chugai Pharma France (Roche group) employee. The remaining authors declare that the research was conducted in the absence of any commercial or financial relationships that could be construed as a potential conflict of interest.

## Publisher's Note

All claims expressed in this article are solely those of the authors and do not necessarily represent those of their affiliated organizations, or those of the publisher, the editors and the reviewers. Any product that may be evaluated in this article, or claim that may be made by its manufacturer, is not guaranteed or endorsed by the publisher.
