# Supplementary material for: Presentation and Real-World Management of Giant Cell Arteritis (Artemis Study)
Source: Front Med (Lausanne). 2021 Nov 11;8:732934. doi: 10.3389/fmed.2021.732934 (PMC8631900; doi:10.3389/fmed.2021.732934)
Supplement: Supplementary file 1 [file Table_1.DOCX]

**Supp Table 1: Diagnostic tests for giant cell arteritis according to the clinical phenotype.**

|  | Cranial manifestations at diagnosis | | PMR symptoms at diagnosis | | Large vessel involvement only | |
| --- | --- | --- | --- | --- | --- | --- |
|  | Test done | Test contributing to diagnosis | Test done | Test contributing to diagnosis | Test done | Test contributing to diagnosis |
| Temporal artery (TA) biopsy | 88.2% | 60.1% | 75.7% | 47.9% | 66.7% | 33.3% |
| High resolution TA doppler | 30.8% | 16.7% | 30.2% | 12.9% | 28.6% | 0 |
| Angio-CT | 28.7% | 8.5% | 22.2% | 9.0% | 46.7% | 40.0% |
| 18FDG-PET | 20.7% | 8.8% | 29.3% | 21.8% | 72.7% | 72.7% |
| MRI of the TA | 7.4% | 3.3% | 5.4% | 0.01% | 0 | 0 |
