# Supplementary material for: Presentation and Real-World Management of Giant Cell Arteritis (Artemis Study)
Source: Front Med (Lausanne). 2021 Nov 11;8:732934. doi: 10.3389/fmed.2021.732934 (PMC8631900; doi:10.3389/fmed.2021.732934)
Supplement: Supplementary file 2 [file Table_2.docx]

**Supp Table 2: Comorbidities related to or aggravated by glucocorticoids use according to the investigator’s judgment (N=306).**

| \| **Comorbidities (≥ 2 patients), n (%)** \| **Total (N=306)** \| \| --- \| --- \| \| **Patients with at least one comorbidity related to or aggravated by glucocorticoids use, n (%)** \| **114 (37.3)** \| \| CARDIAC DISORDERS \| 5 (1.63) \| \| ATRIAL FIBRILLATION \| 3 (0.98) \| \| ENDOCRINE DISORDERS \| 2 (0.65) \| \| EYE DISORDERS \| 9 (2.94) \| \| CATARACT \| 4 (1.31) \| \| GLAUCOMA \| 5 (1.63) \| \| GASTROINTESTINAL DISORDERS \| 4 (1.31) \| \| HEPATOBILIARY DISORDERS \| 2 (0.65) \| \| INFECTIONS AND INFESTATIONS \| 10 (3.27) \| \| INFLUENZA \| 2 (0.65) \| \| INJURY, POISONING AND PROCEDURAL COMPLICATIONS \| 7 (2.29) \| \| SPINAL COMPRESSION FRACTURE \| 2 (0.65) \| \| SPINAL FRACTURE \| 3 (0.98) \| \| INVESTIGATIONS \| 4 (1.31) \| \| WEIGHT INCREASED \| 3 (0.98) \| \| METABOLISM AND NUTRITION DISORDERS \| 44 (14.38) \| \| DIABETES MELLITUS \| 38 (12.42) \| \| DYSLIPIDAEMIA \| 5 (1.63) \| \| HYPERTRIGLYCERIDAEMIA \| 2 (0.65) \| \| MUSCULOSKELETAL AND CONNECTIVE TISSUE DISORDERS \| 25 (8.17) \| \| OSTEOPENIA \| 5 (1.63) \| \| OSTEOPOROSIS \| 14 (4.58) \| \| OSTEOPOROTIC FRACTURE \| 3 (0.98) \| \| NERVOUS SYSTEM DISORDERS \| 7 (2.29) \| \| ESSENTIAL TREMOR \| 2 (0.65) \| \| TRANSIENT ISCHAEMIC ATTACK \| 2 (0.65) \| \| PSYCHIATRIC DISORDERS \| 18 (5.88) \| \| ANXIETY \| 2 (0.65) \| \| DEPRESSION \| 4 (1.31) \| \| INSOMNIA \| 9 (2.94) \| \| RENAL AND URINARY DISORDERS \| 2 (0.65) \| \| RESPIRATORY, THORACIC AND MEDIASTINAL DISORDERS \| 3 (0.98) \| \| SLEEP APNOEA SYNDROME \| 3 (0.98) \| \| SURGICAL AND MEDICAL PROCEDURES \| 4 (1.31) \| \| OSTEOPOROSIS PROPHYLAXIS \| 4 (1.31) \| \| VASCULAR DISORDERS \| 33 (10.78) \| \| AORTIC ANEURYSM \| 2 (0.65) \| \| HYPERTENSION \| 31 (10.13) \| |
| --- | --- | --- | --- | --- | --- | --- | --- | --- | --- | --- | --- | --- | --- | --- | --- | --- | --- | --- | --- | --- | --- | --- | --- | --- | --- | --- | --- | --- | --- | --- | --- | --- | --- | --- | --- | --- | --- | --- | --- | --- | --- | --- | --- | --- | --- | --- | --- | --- | --- | --- | --- | --- | --- | --- | --- | --- | --- | --- | --- | --- | --- | --- | --- | --- | --- | --- | --- | --- | --- | --- | --- | --- | --- | --- | --- | --- | --- | --- | --- | --- |
